# Integrated Serosurveillance for Onchocerciasis, Lymphatic Filariasis, and Schistosomiasis in North Darfur, Sudan

**DOI:** 10.4269/ajtmh.23-0760

**Published:** 2024-06-25

**Authors:** Jenna E. Coalson, Gregory S. Noland, Andrew W. Nute, Erica Brook Goodhew, Diana L. Martin, Zeinab Abdalla, Isam Zarroug, Soheir Gabralla, Hassan Ahmed Hassan Ahmed Ismail, William Evan Secor, Elizabeth Kelly Callahan, Angelia M. Sanders, Balgesa Elshafie, Scott D. Nash

**Affiliations:** ^1^ River Blindness, Lymphatic Filariasis, Schistosomiasis, and Malaria Programs, The Carter Center, Atlanta, Georgia;; ^2^ Trachoma Control Program, The Carter Center, Atlanta, Georgia;; ^3^ Laboratory Science and Diagnostics Branch, Division of Parasitic Diseases and Malaria, U.S. Centers for Disease Control and Prevention, Atlanta, Georgia;; ^4^ Health Programs, The Carter Center, Khartoum, Sudan;; ^5^ Federal Ministry of Health, Khartoum, Sudan

## Abstract

Sudan is endemic for multiple neglected tropical diseases, including trachoma, onchocerciasis (OV), lymphatic filariasis (LF), and schistosomiasis (SCH). In 2019, dried blood spot samples were collected for a baseline trachoma serosurvey in three localities (El Seraif, Kotom, and Saraf Omrah) in North Darfur State. None were classified previously as OV- or LF-endemic, although low levels of SCH had been identified in all three. Approximately 30 households from 25 communities in each locality were selected by multistage cluster random sampling. Collections of DBSs were analyzed by multiplex bead assay for antibodies to multiple pathogens. This paper presents data on OV (Ov16), LF (Wb123, Bm14, Bm33), and SCH (soluble egg antigen [SEA], Sm25) antibodies among 8,322 individuals from 2,119 households. The survey-adjusted seroprevalence estimates for Ov16 were <0.3% in all localities. Lymphatic filariasis–antigen seroprevalences were discordant. Seroprevalence estimates ranged from 4.6–6.0% (Wb123), 0.99–1.4% (Bm14), and 29.2–33.3% (Bm33). Schistosomiasis seroprevalence estimates among school-aged children ranged from 2.7–8.0% (SEA) and 10.9–15.6% (Sm25). Ov16 seropositivity was low and supported the localities’ classification as nonendemic. The results suggested LF exposure, but discordance between antigens, challenges defining seropositivity thresholds, and the absence of programmatic guidance based on antibody serology alone for *Wuchereria bancrofti* indicate a need for remapping surveys to confirm transmission. Schistosomiasis antibody levels were high enough to warrant further mapping to guide treatment decisions. The lack of gold standards limited interpretation of results, particularly for LF, but in resource-challenged areas, integrated serological surveillance offers the possibility of efficient monitoring of exposure to multiple diseases.

## INTRODUCTION

Onchocerciasis (OV), lymphatic filariasis (LF), schistosomiasis (SCH), and trachoma are neglected tropical diseases (NTDs) that disproportionately affect low-resource populations, for which preventive chemotherapy, or mass drug administration (MDA), is a core intervention strategy.^1^ These NTDs require population-based surveys to understand prevalence, intensity, and the need for mitigation and prevention. Both OV and LF are vector-borne filarial diseases that can cause severe morbidity. Onchocerciasis is caused by the filarial parasite *Onchocerca volvulus*, transmitted from person to person by *Simulium* blackflies that breed in fast-moving rivers. Chronic infection with *O. volvulus* results in skin disease and potential blindness, leading to the common name “river blindness.” Lymphatic filariasis is caused by the parasites *Wuchereria bancrofti, Brugia malayi,* or *Brugia timori*, transmitted by mosquitoes from several genera. Chronic infection damages the lymphatic vessels and can lead to severe disability and disfiguration from hydrocele, lymphedema, and the most advanced form of lymphedema, elephantiasis.^2^ Schistosomiasis is caused by helminths of the genus *Schistosoma*, is transmitted through water, and can cause either intestinal or urogenital forms of illness.^3^ Symptoms of the intestinal form of SCH include abdominal pain, diarrhea, pulmonary hypertension, and a range of other symptoms. Urogenital infection can lead to hematuria, dysuria, and infertility. Within Africa, the most common SCH species are *Schistosoma mansoni* and *Schistosoma haematobium*.^4^ Global strategies to eliminate transmission of OV, to eliminate LF as a public health problem, and to control SCH include mass provision of drugs to populations in endemic areas.^1^ Drug treatments (ivermectin alone for OV and albendazole coadministered with ivermectin and/or diethylcarbamazine for LF) kill embryonic forms of the parasites called microfilariae that are ingested by blood-feeding blackflies or mosquitoes, respectively.^5^^,^^6^ Praziquantel is effective in killing adult schistosomes but is less effective against larval forms and does not prevent reinfection. The WHO NTD Road Map 2021–2023 has targeted elimination of OV transmission in 12 countries by 2030, elimination of LF as a public health problem in 58 countries by 2030, and validation of elimination of SCH as a public health problem in all 78 endemic countries by 2030.^7^

Sudan is endemic for multiple NTDs including OV, LF, and SCH. Onchocerciasis in Sudan is historically described in four transmission foci: Abu Hamad in River Nile State in northern Sudan, Galabat in Gedaref State bordering Ethiopia to the East, Khor Yabus in Blue Nile State bordering Ethiopia and the Republic of South Sudan, and Radom in South Darfur State bordering the Republic of South Sudan and Central African Republic.^8^ The provisional serological threshold for starting MDA for OV is Ov16 IgG4 antibody prevalence greater than 2% in adult residents of first-line villages,^9^ whereas the threshold for stopping MDA is an Ov16 seroprevalence with an upper 95% confidence limit of less than 0.1% in children 5–9 years old using an ELISA platform in addition to entomological data.^10^

Lymphatic filariasis in Africa is caused by *W. bancrofti,* and as of 2019, Sudan is the only country in the WHO Eastern Mediterranean Region with ongoing LF transmission.^11^ The Sudanese Federal Ministry of Health (FMOH) conducted nationwide LF mapping surveys at a purposively selected, high-risk sentinel site in each locality (i.e., district) in 2016. Based on a WHO threshold of circulating filarial antigen (CFA) prevalence greater than or equal to 1% among adults older than 15 years, LF was initially considered endemic in 61 (32.4%) of the then 185 localities.^11^ The threshold for stopping MDA for *W. bancrofti* is a CFA prevalence of less than 2% among children 6–7 years old in *Anopheles* or *Culex* mosquito transmission areas.^12^

Between 2016 and 2017, a nationwide SCH survey was conducted among schoolchildren in Sudan using parasitological techniques (Kato Katz to examine *S. mansoni* eggs in stool samples and the centrifugation method to examine *S. haematobium* eggs in urine samples).^13^ *Schistosoma haematobium* was far more prevalent and widespread than *S. mansoni*, and the highest prevalence of *S. haematobium* infections was found in East and South Darfur States in the southwestern part of the country.^13^ Mapping and impact assessments rely on parasitological assessments for prevalence and intensity of infections. The WHO guidelines from 2011 recommended school-based MDA with the frequency dependent on prevalence, though newly released 2022 guidelines recommended a shift to community-based MDA strategies (2 years and older).^14^^,^^15^

Mapping of endemicity and impact assessments are key steps for disease elimination programs, but these activities can be resource intensive. Therefore, NTD programs are increasingly exploring integrated assessments, including multi-analyte serosurveillance using multiplex bead assay (MBA).^16^^,^^17^ This paper presents the results of an integrated assessment for NTDs in North Darfur State, Sudan with specific focus on Ov16 antigen for OV, Wb123, Bm14, and Bm33 antigens for LF, and soluble egg antigen (SEA) and Sm25 antigens for SCH, linked to nonspecific SCH exposure and *S. mansoni*–specific exposure, respectively. Data collection was originally undertaken as part of a trachoma baseline survey; analyses of the trachoma results are reported in a companion article.^18^

## MATERIALS AND METHODS

### Study setting.

**Figure 1.**
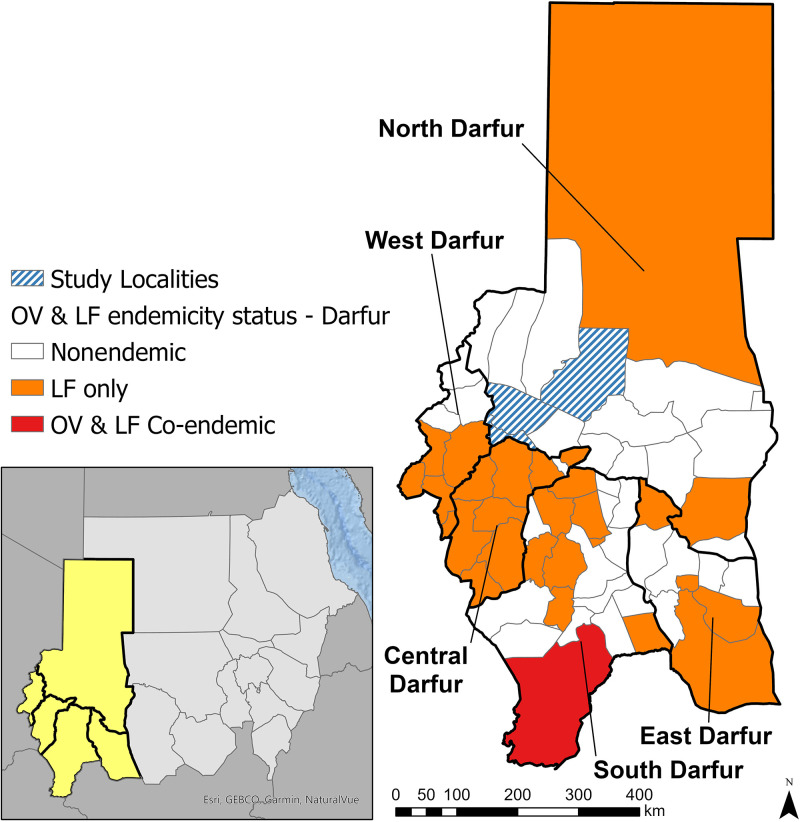
Study locations and endemicity for onchocerciasis (OV) and lymphatic filariasis (LF) by locality, Darfur region, Sudan.

This study was conducted in three localities (the sub-state administrative unit, similar to a district) in North Darfur State, part of the Darfur region in western Sudan: El Seraif, Kotom, and Saraf Omrah (Figure 1). North Darfur is in the Sahel, experiencing an average annual rainfall of only 176 mm,^19^ and the study localities do not have any major rivers. They are not considered OV endemic and are more than 300 km from the nearest known OV transmission focus in Radom. The study localities were also classified as nonendemic for LF based on the absence of CFA-positive individuals in the sentinel site selected during the 2016 mapping effort, but each locality borders an LF-endemic locality in neighboring North, Central, or West Darfur States (Figure 1). Based on the 2016–2017 nationwide SCH survey, the estimated prevalence of *S. haematobium* infection among school-aged children (SAC) was 1.9% in El Seraif, 0.7% in Kotom, and 10.3% in Saraf Omrah.^13^ The prevalence of *S. mansoni* infection in this age group was 0.4%, 0.3%, and 0.8% in El Seraif, Kotom, and Saraf Omrah, respectively.^13^ Since the nationwide survey, there has been no record of programmatic implementation of SCH interventions in North Darfur. Because the three localities have been considered nonendemic for OV and LF, they are also ivermectin and albendazole MDA–naive.

### Study design.

The primary purpose of the survey was to map trachoma endemicity in the three localities using a cross-sectional survey conducted in late 2019 and early 2020.^18^ The Sudan FMOH and partners leveraged this trachoma survey to collect seroprevalence data on other diseases of public health importance as described below, providing these results at no additional cost to other disease programs.

### Sampling and enrollment.

In each of the three study localities, multistage cluster random sampling was used to select the study population. In the first stage, 30 clusters (communities) were selected randomly from a geographically ordered list provided by the State Ministry of Health Expanded Program on Immunization. In the second stage, community leaders were asked to prepare maps and community lists of all households within the clusters for segment sampling. Households were then grouped into segments of five, from which five segments were randomly selected, giving a total of 25 households from each cluster. All individuals in each household who were at least 1 year of age were eligible for enrollment in the study.

Sample size targets were designed based on the primary study goals of the trachoma program to estimate a baseline trachomatous inflammation–follicular prevalence of 10% among children aged 1–9 years with a precision of 3%. Calculations assumed a design effect of 2.65, 95% CIs, a nonresponse rate of 20%, an average of 4.7 individuals per household, and that children 1–9 years old comprised 35% of the population. Based on these assumptions, roughly 743 households with 1,222 children were targeted for each locality.

### Data collection.

This study used electronic data collection, with surveys completed on phones or tablets. Survey data were collected using Open Data Kit Collect to submit XML forms to NEMO (getnemo.org), The Carter Center’s open-source data collection and reporting system. Global Positioning System coordinates were taken for each survey cluster. The female head of household (if available) was interviewed to provide information on household-level data, after which household members were enumerated and screened for signs of trachoma.

Trained nurses collected DBSs from finger prick samples from consenting/assenting individuals at least 1 year of age from the selected households. Filter papers were air-dried for at least 2 hours and stored in Ziploc bags with desiccant at −20°C until flown at ambient temperature to the U.S. Centers for Disease Control and Prevention, Atlanta, GA, for MBA testing.

## STATISTICAL ANALYSES

For total IgG antibody responses to 19 various antigens from 11 pathogens of public health importance, DBSs were assayed by MBA. Antigen-coupled beads were added to 96-well filter-bottom plates (Millipore, Bedford, MA) and were washed twice with phosphate-buffered saline plus 0.05% Tween-20 (PBST). Control sera and bloodspot eluates (1:400) were then added, beads were suspended and protected from light, and the plates were shaken on a plate shaker at room temperature for 1.5 hours. After the beads were washed three times with 100 *µ*L PBST, total IgG was detected with 50 ng of biotinylated mouse anti-human total IgG (clone H2; Southern Biotech, Birmingham, AL) and 40 ng of biotinylated mouse anti-human IgG4 (clone HP6025; Invitrogen, South San Francisco, CA) per well in 50 *µ*L assay buffer (1× PBS [phosphate-buffered saline], 0.5% BSA [bovine serum albumin], 0.02% NaN_3_, and 0.05% Tween-20). After a second wash step, streptavidin-phycoerythrin (SAPE; Invitrogen, South San Francisco, CA) was added at a concentration of 250 ng per well in assay buffer and incubated for 30 minutes at room temperature. After washing to remove SAPE, any loosely bound antibodies were removed with an additional incubation in assay buffer. After a final wash in PBST, beads were suspended in 100 *µ*L PBS and stored overnight at 4°C. The following day, plates were shaken and read on a Luminex instrument (Luminex Corp., Austin, TX) equipped with Bio-Plex Manager 6.0 software (Bio-Rad, Hercules, CA).

This report presents the findings for OV antigen Ov16^20^^–^^22^; LF antigens Wb123,^23^^–^^25^ Bm14, and Bm33^26^^–^^29^; SEA for nonspecific exposure to any of the four Africa-endemic species that can cause human SCH; and Sm25 for exposure to *S. mansoni*.^30^^–^^33^ Median fluorescence intensities minus background (MFI-BG) from study subjects were compared against those of 86 U.S.-resident, non–overseas traveling adult controls for all antibodies. Subjects classified as seropositive for Ov16, Wb123, Bm14, and Bm33 antibodies were those who had MFI-BG values of at least 3 SDs above the mean MFI-BG values for the nontravelers. The thresholds for seropositivity for the schistosome antigens were calculated from a receiver operating characteristic curve involving the aforementioned 86 nontravelers combined with a WHO panel of 37 negative and 117 *S. mansoni*– or *S. haematobium*–positive controls for SEA or 72 *S. mansoni*–positive controls for Sm25. The MFI-BG threshold for Ov16 was 1,315; for Wb123 it was 232; for Bm14 it was 838; for Bm33 it was 385; for Sm25 it was 73; and for SEA it was 117.

Data were cleaned and analyzed using R version 4.2.2 and Stata v. 17.0 (StataCorp LLC., College Station, TX). Given the complex sampling design, estimated seroprevalence, Pearson χ^2^ tests for differences in seroprevalence by sex, and *t*-tests for differences in seroprevalence by age were calculated using the svy command in Stata procedures to account for survey weights. Age-specific results for SCH were reported for preschool-aged children (PSAC; 1–4 years), SAC (5–14 years), and adults (15+ years) based on the traditional importance of SAC in SCH monitoring and treatment guidelines.^14^ Reversible catalytic models were fit to the seropositivity by age for each antigen by locality using a simple model by maximum likelihood to estimate the serological conversion and reversion rates, which were assumed to be constant over time. These were calculated using the REVCAT Stata module, with results presented in Supplemental File 1.^34^ Concordance between seropositivity to the two SCH antigens (Sm25 and SEA) was evaluated using Cohen’s κ, and between the three LF antigens (Wb123, Bm33, and Bm14), it was assessed pairwise by Cohen’s κ and across all three antigens by Fleiss’s κ.^35^^,^^36^ Maps were created, and spatial analysis was conducted in ArcGIS Pro 3.0.2 (Esri, Redlands, CA). We tested for spatial autocorrelation of village seroprevalence to each antigen using Global Moran’s I with inverse weighting of distances.

### Ethical considerations.

The studies reported here were approved by the institutional review boards (IRBs) of Emory University and the Sudanese FMOH as activities undertaken for program monitoring and evaluation. The survey and methodology were explained to each participant, who was then given the opportunity to ask questions and agree to or decline participation. Because of illiteracy among the population, IRB approval was obtained for verbal consent. Staff from the U.S. CDC did not have contact with study participants or access to identifying information and were determined to be not engaged in research on human subjects.

## RESULTS

A total of 8,324 individuals provided DBSs; the final study population comprised 8,322 subjects for whom valid MBA results were available from 2,119 households across the three localities, with a mean of 3.9 people per household (Table 1). Participants tended to be young, with children less than age 20 years comprising more than 60% of the sampled population. Of the 8,322 people, 3,674 (44.1%) were children under the age of 10 years. More women were included in the sample than men, with women comprising ∼60% of participants in all three localities. The sex distribution was approximately even among children less than 10 years of age (50.8% female, 49.2% male) and highly discrepant for young and middle-aged adults, 20–50 years of age, where the ratio of women-to-men was 3:1. These age and sex distributions were similar in all three localities. The frequency of seropositivity to Ov16, each of the three LF-associated antigens, and the two SCH-associated antigens are reported by age category, sex, and locality in Table 2. The distributions of MFI-BG values for all subjects and antigens are presented in Supplemental File 2.

**Table 1:** Characteristics of participants by locality, North Darfur cross-sectional study 2019–2020

Characteristics	El Seraif *n* (%)	Kotom *n* (%)	Saraf Omrah *n* (%)	Total *n* (%)
Households	655	804	660	2,119
People Sampled per Household, mean (SD)	4.3 (2.3) Range: 1–15	3.5 (1.9) Range: 1–11	4.1 (2.2) Range: 1–12	3.9 (2.2) Range: 1–15
Highest Education Level of Any Adult in the Household				
None	148 (22.9%)	62 (7.8%)	260 (39.6%)	470 (22.4%)
Primary	275 (42.5%)	384 (48.4%)	295 (45.0%)	954 (45.5%)
Secondary	55 (8.5%)	186 (23.5%)	48 (7.3%)	289 (13.8%)
College/University	33 (5.1%)	125 (15.8%)	18 (2.7%)	176 (8.4%)
Religious Education	134 (20.7%)	34 (4.3%)	35 (5.3%)	203 (9.7%)
Adult Literacy	2 (0.3%)	2 (0.3%)	0 (0%)	4 (0.2%)
Individuals	2,848	2,781	2,693	8,322
Sex				
Women	1,680 (59.0%)	1,699 (61.1%)	1,643 (61.0%)	5,022 (60.3%)
Men	1,168 (41.0%)	1,082 (38.9%)	1,050 (39.0%)	3,300 (39.7%)
Age (in years)				
1–9	1,275 (44.8%)	1,127 (40.5%)	1,272 (47.2%)	3,674 (44.1%)
10–19	627 (22.0%)	483 (17.4%)	554 (20.6%)	1,664 (20.0%)
20–29	326 (11.4%)	295 (10.6%)	295 (11.0%)	916 (11.0%)
30–39	236 (8.3%)	269 (9.7%)	201 (7.5%)	706 (8.5%)
40–49	139 (4.9%)	172 (6.2%)	139 (5.2%)	450 (5.4%)
50–59	85 (3.0%)	132 (4.7%)	77 (2.9%)	294 (3.5%)
≥60	160 (5.6%)	303 (10.9%)	155 (5.8%)	618 (7.4%)

**Table 2:** Seropositivity for onchocerciasis-, lymphatic filariasis–, and schistosomiasis-related antibodies by locality per multiplex bead array assay, North Darfur Cross-sectional Survey, 2019–2020

Variables	El Seraif			Kotom			Saraf Omrah		
	*n*+/*n* tested	Seroprevalence (95% CI)	*P*-Value*	*n*+/*n* tested	Seroprevalence (95% CI)	*P*-Value*	*n*+/*n* tested	Seroprevalence (95% CI)	*P*-Value*
Ov16 (Onchocerciasis)									
Total	5/2,848	0.12% (0.04–0.33%)	–	4/2,781	0.15% (0.04–0.58%)	–	7/2,693	0.26% (0.11–0.63%)	–
Sex									
Women	2/1,680	0.07% (0.02–0.32%)	–	2/1,699	0.10% (0.02–0.50%)		4/1,643	0.23% (0.08–0.67%)	–
Men	3/1,168	0.19% (0.05–0.67%)	0.31	2/1,082	0.23% (0.06–0.90%)	0.17	3/1,050	0.32% (0.10–0.99%)	0.60
Age (in years)^†^									
1–4	2/576	0.14% (0.02–0.89%)	–	1/538	0.17% (0.02–1.25%)	–	0/545	0% (n/a)	–
5–9	3/699	0.37% (0.11–1.19%)	–	1/589	0.24% (0.04–1.62%)	–	4/727	0.51% (0.17–1.50%)	–
10–19	0/627	0% (n/a)	–	1/483	0.32% (0.05–2.1%)		0/554	0% (n/a)	
Adults ≥20	0/946	0% (n/a)	**<0.001**	1/1,171	0.03% (0.00–0.22%)	0.07	3/867	0.40% (0.13–1.22%)	0.24
Wb123 (LF)									
Total	112/2,848	3.6% (2.7–4.9%)	–	104/2,781	3.6% (2.8–4.6%)	–	87/2,693	3.7% (2.8–4.9%)	–
Sex									
Women	63/1,680	3.6% (2.4–5.3%)	–	66/1,699	3.8% (2.7–5.4%)	–	52/1,643	3.9% (2.7–5.6%)	–
Men	49/1,168	3.7% (2.7–4.9%)	0.88	38/1,082	3.2% (2.4–4.3%)	0.48	35/1,050	3.4% (2.6–4.5%)	0.52
Age (in years)									
1–9	38/1,275	2.7% (1.9–3.7%)	–	33/1,127	3.1% (2.2–4.2%)	–	28/1,272	2.5% (1.6–3.9%)	–
10–19	18/627	3.4% (1.1–9.9%)	–	14/483	2.2% (1.2–4.1%)	–	18/554	3.0% (1.9–4.9%)	–
Adults ≥20	56/946	5.1% (3.7–6.9%)	**0.02**	57/1,171	4.6% (3.2–6.6%)	0.28	41/867	6.0% (4.2–8.6%)	**0.002**
Bm14 (LF)									
Total	26/2,848	0.86% (0.48–1.53%)	–	23/2,781	1.04% (0.63–1.70%)	–	22/2,693	0.85% (0.56–1.28%)	–
Sex									
Women	18/1,680	0.75% (0.40–1.4%)	–	17/1,699	1.1% (0.67–1.9%)	–	17/1,643	1.1% (0.66–1.8%)	–
Men	8/1,168	1.0% (0.40–2.5%)	0.57	6/1,082	0.87% (0.36–2.1%)	0.57	5/1,050	0.47% (0.19–1.2%)	0.13
Age (in years)									
1–9	8/1,275	0.73% (0.34–1.6%)	–	5/1,127	0.42% (0.16–1.1%)	–	7/1,272	0.39% (0.18–0.85%)	–
10–19	9/627	0.92% (0.40–2.1%)	–	7/483	1.6% (0.66–3.9%)	–	6/554	1.3% (0.60–2.6%)	–
Adults ≥20	9/946	0.99% (0.42–2.3%)	0.29	11/1,171	1.4% (0.68–3.0%)	0.11	9/867	1.2% (0.61–2.5%)	0.18
Bm33 (LF)									
Total	761/2,848	25.7% (22.4–29.4%)	–	666/2,781	23.7% (21.7–25.9%)	–	721/2,693	29.0% (25.3–33.0%)	–
Sex									
Women	463/1,680	27.3% (23.4–31.5%)	–	429/1,699	25.0% (22.7–27.4%)	–	463/1,643	30.1% (26.2–34.3%)	–
Men	298/1,168	23.4% (19.4–28.0%)	0.11	237/1,082	21.7% (18.5–25.3%)	0.11	258/1,050	27.1% (22.7–32.1%)	0.16
Age (in years)									
1–9	269/1,275	20.5% (17.4–23.9%)	–	205/1,127	18.0% (15.6–20.8%)	–	296/1,272	25.3% (20.8–30.3%)	–
10–19	179/627	25.6% (20.0–32.1%)	–	120/483	24.3% (20.6–28.5%)	–	153/554	30.6% (24.6–37.3%)	–
Adults ≥20	313/946	33.2% (28.8–37.9%)	**<0.001**	341/1,171	29.2% (26.0–32.6%)	**<0.001**	272/867	33.3% (28.7–38.4%)	**0.002**
SEA (Schistosomiasis)									
Total	319/2,848	10.8% (8.3–13.9%)	–	118/2,781	4.2% (3.3–5.4%)	–	196/2,693	7.3% (5.8–9.2%)	–
Sex									
Women	119/1,680	6.2% (4.6–8.3%)	–	50/1.699	2.9% (2.2–3.8%)	–	88/1,643	5.6% (3.9–8.1%)	–
Men	200/1,168	17.7% (12.9–23.7%)	**<0.001**	68/1.082	6.5% (4.7–8.9%)	**<0.001**	108/1,050	10.0% (8.3–12.1%)	**0.002**
Age (in years)^†^									
PSAC 1–4	12/576	1.6% (0.70–3.4%)	–	12/538	3.0% (1.6–5.8%)	–	5/545	0.87% (0.37–2.0%)	–
SAC 5–14	94/1,124	8.0% (5.9–10.8%)	–	26/923	2.7% (1.7–4.2%)	–	45/1,082	4.5% (3.1–6.5%)	–
Adults 15+	213/1,148	18.5% (13.7–24.4%)	**<0.001**	80/1.320	5.9% (4.5–7.6%)	**<0.001**	146/1,066	13.5% (10.6–17.2%)	**<0.001**
Sm25 (*S. mansoni*)									
Total	449/2,848	15.6% (11.9–20.2%)	–	453/2,781	16.1% (13.6–18.8%)	–	369/2,693	17.2% (12.8–22.8%)	–
Sex									
Women	272/1,680	16.8% (12.9–21.5%)	–	312/1,699	18.0% (15.0–21.4%)	–	225/1,643	17.0% (12.4–22.7%)	–
Men	177/1,168	13.9% (10.1–18.9%)	**0.04**	141/1,082	12.9% (10.6–15.5%)	**<0.001**	144/1,050	17.7% (13.0–23.6%)	0.62
Age (in years)^†^									
PSAC 1–4	15/576	2.4% (1.2–4.9%)	–	13/538	2.0% (1.1–3.8%)	–	15/545	3.3% (1.5–6.9%)	–
SAC 5–14	144/1,124	13.3% (10.1–17.3%)	–	96/923	10.9% (8.3–14.1%)	–	122/1,082	15.6% (10.6–22.5%)	–
Adults 15+	290/1,148	25.0% (18.9–32.3%)	**<0.001**	344/1,320	25.9% (22.0–30.2%)	**<0.001**	232/1,066	25.9% (20.5–32.3%)	**<0.001**

LF = lymphatic filariasis; n/a = not applicable; PSAC = preschool-aged children; *S. mansoni* = *Schistosoma mansoni*; SAC = school-aged children; SEA = soluble egg antigen. Bolded *P*-values indicate statistical significance at <0.05.

* *P*-values were calculated from χ^2^ tests for sex and *t*-tests for age as a continuous variable, accounting for the survey weights using svy commands in Stata.

^†^ Age groups were reported differently depending on groupings that were relevant for the different disease interventions/studies.

### Onchocerciasis seroprevalence.

A total of only 16 individuals tested positive for Ov16 antibodies (Table 2). Locality-specific weighted seroprevalence estimates were 0.12% (95% CI: 0.04–0.33%) in El Seraif, 0.15% (95% CI: 0.04–0.58%) in Kotom, and 0.26% (95% CI: 0.11–0.63%) in Saraf Omrah. The associated seroconversion and seroreversion rates were imprecise given the low numbers and lack of clear age patterns (Supplemental File 1).

**Figure 2.**
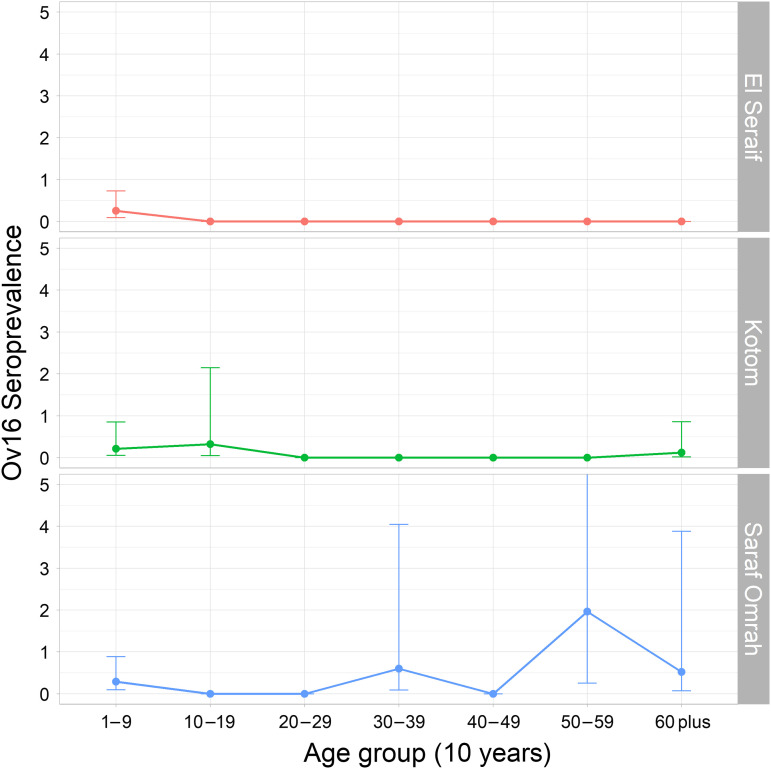
Seroprevalence of Ov16 by multiplex bead array assay by 10-year age group in three localities of North Darfur, 2019–2020. Note: The upper 95% confidence limit of the seroprevalence for the 50–59-year age group in Saraf Omrah extends to 13.6%. Error bars represent 95% confidence intervals.

The Ov16 seroprevalence estimates of men were consistently higher than those of women in each of the three localities, though the differences were not statistically significant (overall *P* = 0.16). There was no statistical evidence of geographic autocorrelation (Supplemental File 3; Moran’s I = −0.04, *Z*-score = −0.46, *P* = 0.65) or increasing antibody positivity with age (Table 2; Figure 2). In fact, seroprevalence estimates were lowest in adults in both El Seraif and Kotom. Among 2,984 adults at least 20 years old, there were only four who tested positive for Ov16, three of whom were from Saraf Omrah. The estimated seroprevalence among adults in Saraf Omrah was 0.40% (95% CI: 0.13–1.22%), well beneath the WHO start-MDA threshold of 2%.^9^ Seroprevalence was highest in children 5–9 years old, with eight of the study’s 16 seropositive individuals in this age group.

### Lymphatic filariasis seroprevalence.

**Figure 3.**
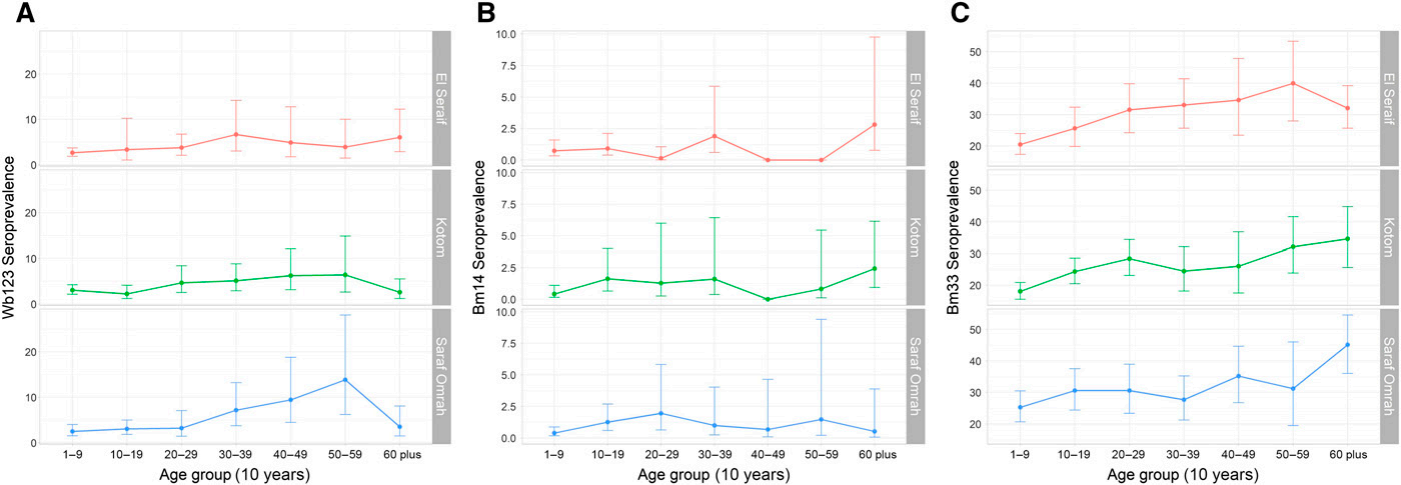
Seropositivity by locality and 10-year age group in North Darfur, 2019–2020, for lymphatic filariasis–associated antigens, (**A**) Wb123, (**B**) Bm14, and (**C**) Bm33, by multiplex bead array assay. Note that the *y* axis differs for each antigen. Error bars represent 95% confidence intervals.

The weighted estimates of seroprevalence against Wb123 were consistent across the three localities at 3.6% (95% CI: 2.7–4.9%) in El Seraif, 3.6% (95% CI: 2.8–4.6%) in Kotom, and 3.7% (95% CI: 2.8–4.9%) in Saraf Omrah (Table 2). The seroprevalence tended to increase with age (Table 2; Figure 3A), with the increase being statistically significant in El Seraif (*P* = 0.02) and Saraf Omrah (*P* = 0.002). There was no statistically significant evidence of spatial autocorrelation of village Wb123 seroprevalence (Supplemental File 4A; Moran’s I = 0.07, *Z*-score = 1.10, *P* = 0.27).

The estimated seroprevalence of Bm14 was lower than that of Wb123, ranging from 0.85% in Saraf Omrah to 1.04% in Kotom, but the estimated seroprevalence of Bm33 was much higher than the other two antibodies, ranging from 23.7% in Kotom to 29.0% in Saraf Omrah (Table 2). An increasing pattern of seropositivity with age was observed for Bm33 but not for Bm14 (Table 2; Figure 3B and C). There was no statistically significant evidence of spatial autocorrelation of village Bm14 seroprevalence (Supplemental File 4B; Moran’s I = −0.11, *Z*-score = −1.32, *P* = 0.19) or Bm33 seroprevalence (Supplemental File 4C; Moran’s I = 0.05, *Z*-score = 0.78, *P* = 0.44).

**Figure 4.**
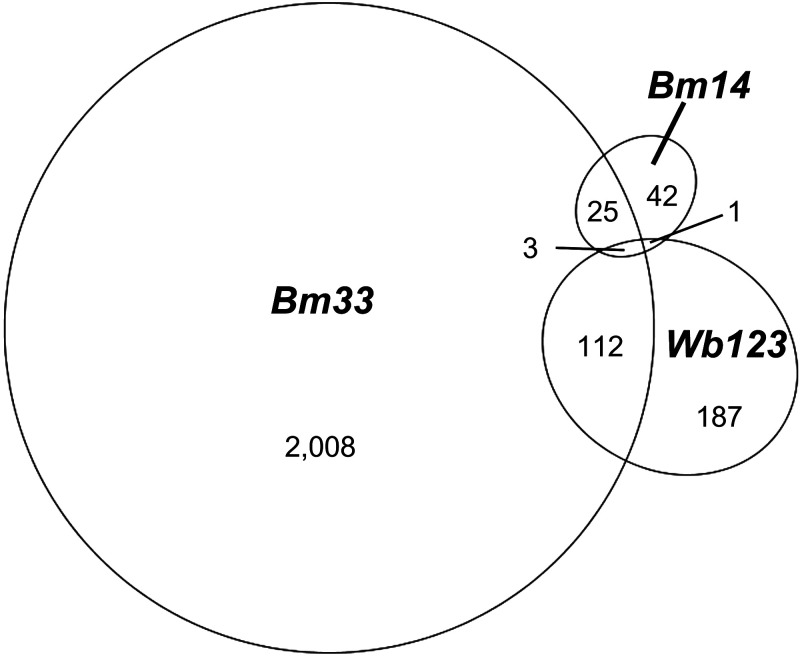
Correspondence in seropositivity for three lymphatic filariasis antigens, *N* = 8,322, North Darfur, Sudan, 2019–2020. Created using eulerAPE.^37^

Seropositivity to the three LF-associated antigens was highly discordant in this dataset (Figure 4). Only three individuals of the 8,322 in the population tested positive to all three of the antigens, and the Fleiss’s κ for the concordance of all three antigens was less than chance at −0.05. The likelihood of testing positive to Bm33 was significantly associated with the likelihood of testing positive to Wb123, with the Bm33 seroprevalence being 38.7% among people seropositive for Wb123 versus 25.6% among people seronegative for Wb123 (*P* = 0.0001); however, 61.7% (187 of 303) of Wb123 seropositive samples did not test positive for either Bm14 or Bm33. The observed concordance between Bm33 and Wb123 serostatus was 73.3% versus an expected concordance of 72.4%, resulting in a κ value of only 0.03 (*P* <0.001), reflecting a statistically significant but weak concordance beyond chance. The same was true for the concordance of Bm33 and Bm14 serostatus (observed concordance 74.0% versus expected concordance of 73.8%, κ = 0.003, *P* = 0.004). The concordance between Bm14 and Wb123 was high at 95.6%, but this was not significantly better than the expected concordance by random chance of 95.6%, with a κ value of only 0.008 (*P* = 0.18).

Many subjects deemed seropositive, especially for Wb123 and Bm33, had MFI-BG values close to the threshold (Supplemental File 2), highlighting the challenges of accurate binary classification of seropositivity for LF and the high risks of false-positives and false-negatives depending on threshold selection. Poor specificity may be ameliorated by composite outcomes requiring positive responses to multiple antigens; seroprevalence estimates for various LF antigen combinations are available in Supplemental File 5. When requiring that participants be positive to all three antigens to be considered LF seropositive, the seroprevalence estimates dropped to 0.04% for El Seraif, 0% for Kotom, and 0.04% for Saraf Omrah. When requiring that positivity to at least two of any of the three antigens, the LF seroprevalence estimates would be 3.4%, 2.7%, and 1.9%, respectively. No gold standard diagnostic was available to evaluate the best algorithm for determining LF seroprevalence in this context.

Because Bm14 and Bm33 are known to cross-react with other filarial parasites, we evaluated whether ownership of livestock, as a proxy for exposure to zoonotic filarial diseases, was associated with seropositivity to Bm33 in this population. No statistically significant difference was found for either Bm33 (*P* = 0.31) or Bm14 (*P* = 0.98), with a Bm33 seroprevalence at 27.2% and Bm14 seroprevalence at 0.92% among households that did not own livestock versus 25.6% and 0.93%, respectively, among those in households that did.

### Schistosomiasis seroprevalence.

**Figure 5.**
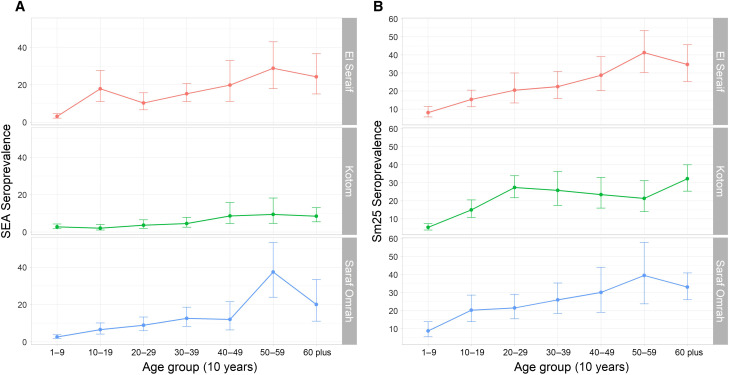
Seropositivity by locality and 10-year age group in North Darfur, 2019–2020, for schistosomiasis-associated antigens (**A**) SEA and (**B**) Sm25 by multiplex bead assay. Error bars represent 95% confidence intervals.

The weighted estimates of seroprevalence against SEA were 10.8% (95% CI: 8.3–13.9%) in El Seraif, 4.2% (95% CI: 3.3–5.4%) in Kotom, and 7.3% (95% CI: 5.8–9.2%) in Saraf Omrah. Soluble egg antigen seroprevalence was statistically significantly (*P* <0.01) higher among men than women in all three localities. The estimated seroprevalence against SEA among SAC was 8.0% (95% CI: 5.9–10.8%) in El Seraif, 2.7% (95% CI: 1.7–4.2%) in Kotom, and 4.5% (95% CI: 3.1–6.5%) in Saraf Omrah. Among PSAC, the highest prevalence against SEA was observed in Kotom (3.0%). The increase in seroprevalence with age was statistically significant (*P* <0.001) for all three localities (Table 2; Figure 5A). There was significant evidence of clustering overall (Moran’s I = 0.30, *Z*-score = 4.06, *P* <0.0001) and in SAC (Moran’s I = 0.14, *Z*-score = 1.97, *P* = 0.05), with high-seroprevalence villages tending to fall in the southwest in El Seraif and Saraf Omrah (Supplemental File 6A and B).

The weighted estimates of seroprevalence against Sm25 were generally higher than those of SEA and were consistent across the three localities at 15.6% (95% CI: 11.9–20.2%) in El Seraif, 16.1% (95% CI: 13.6–18.8%) in Kotom, and 17.2% (95% CI: 12.8–22.8%) in Saraf Omrah (Table 2). Among SAC, the seroprevalence was 13.3% (95% CI: 10.1–17.3%) in El Seraif, 10.9% (95% CI: 8.3–14.1%) in Kotom, and 15.6% (95% CI: 10.6–22.5%) in Saraf Omrah. Among PSAC, the highest prevalence against Sm25 was observed in Saraf Omrah (3.3%). An increasing pattern of seropositivity with age (*P* <0.001) was observed for this antigen, reaching a high of 26.4% prevalence among adults in Saraf Omrah (Table 2; Figure 5B). The Sm25 seroprevalence was somewhat geographically clustered in southern Saraf Omrah, southern El Seraif, and southeast Kotom (Supplemental File 6C and D). This spatial clustering was of borderline significance in all-age seroprevalence (Moran’s I = 0.13, *Z*-score = 1.81, *P* = 0.07) and was more strongly evident in SAC (Moran’s I = 0.17, *Z*-score = 2.43, *P* = 0.02). The concordance between the two antigens was 82.3% overall compared with an expected concordance of 79.4%, giving a κ value of only 0.14 (Table 3). The κ values rating concordance between the two antigens beyond the random expected values were highest in Saraf Omrah (84.1% versus expected of 81.0%, κ = 0.16) and among adults (73.7% versus expected of 69.2%, κ = 0.14) compared with SAC (84.8% versus expected of 84.4%, κ = 0.024) and PSAC (95.8% versus expected of 95.8%, κ = 0.007).

**Table 3:** Concordance between seropositivity to schistosomiasis antigens Sm25 and SEA, North Darfur, Sudan, 2019–2020

Population/Subpopulation	Sm25 Serostatus	SEA Serostatus		Observed Concordance	Expected Concordance	Cohen’s κ	*P*-Value
		-	+				
Total	-	6,632	419	82.3%	79.4%	0.14	<0.0001
	+	1,057	214				
Locality: El Seraif	-	2,177	222	79.9%	76.6%	0.14	<0.0001
	+	352	97				
Locality: Kotom	-	2,258	70	82.9%	80.9%	0.11	<0.0001
	+	405	48				
Locality: Saraf Omrah	-	2,197	127	84.1%	81.0%	0.16	<0.0001
	+	300	69				
Age: PSAC 1-4 years	-	1,588	28	95.8%	95.8%	0.007	0.38
	+	42	1				
Age: SAC 5-14 years	-	2,627	140	84.8%	84.4%	0.024	0.07
	+	337	25				
Age: Adults 15+ years	-	2,417	251	73.7%	69.2%	0.14	<0.0001
	+	678	188				

## DISCUSSION

Use of MBA technology during a trachoma survey in North Darfur State, Sudan provided serological data for several other NTDs, including OV, LF, and SCH, at no additional cost to those programs. To our knowledge, this is the first survey to apply MBA for epidemiological data collection for multiple diseases in one platform in Sudan and the first published report of OV epidemiological assessments by MBA in any country. This tool has promise for cost-effective monitoring in hard-to-reach settings such as North Darfur, though research and guidance are needed to overcome several limitations affecting the interpretability of the currently used antigens for OV, LF, and SCH.

Serological monitoring offers many advantages for resource-poor settings such as North Darfur. Multiple pathogens can be analyzed from a single DBS sample obtained by fingerstick. This avoids collection of skin snip, stool, and/or urine specimens. Dried blood spots can be stored as long as needed under appropriate conditions until testing is conducted. Further, DBS collection allows for integrated serosurveillance, an approach that is becoming more common.^16^^,^^38^^,^^39^ Embedding DBS collection into a trachoma survey allowed for the collection of data on responses to multiple antigens from a community-based sample of participants of all ages. This integrated serological monitoring enables cost efficiencies.

The sampling was optimized for trachoma rather than for OV, LF, and SCH, with the target sample size calculated based on the desired precision of the prevalence estimate for children aged 1–9 years. Although specific guidelines are still being refined for the elimination context, endemicity mapping for OV, LF, and SCH has typically involved sampling from a few purposively selected high-risk communities per implementation unit, targeting 50–100 adults 20 years and older in three to five villages for OV, 50–100 adults 15 years and older in one or two villages for LF, and 50 SAC 10–14 years old in five villages for SCH.^40^^–^^43^ Because the trachoma survey sampled all individuals over 1 year of age in cluster-randomly selected households, meaningfully large sample sizes were achieved in the age groups relevant to mapping for OV, LF, and SCH, but high-risk communities were not purposively sampled. In this region of North Darfur, where there was no prior evidence for high-risk villages for OV and LF, random sampling of communities/clusters was nonetheless reasonably informative. Attention may need to be paid to ensuring that high-risk communities are included in the sample if trachoma surveys are used as the basis to inform OV, LF, and SCH mapping in other areas in the future.

Key limitations of these data on OV, LF, and SCH antigens were the lack of WHO guidelines on programmatic actions based on MBA serology and the absence of comparative results from accepted programmatic tests for OV, LF, and SCH run in parallel. No WHO recommendations exist for *W. bancrofti* or SCH control programs based on serology generally or serology by MBA in particular. Although there are guidelines for OV serology, they are based on IgG4 detection. It is not clear if the highly sensitive MBA detection of total IgG is suitably specific for decision-making in low- and nonendemic OV and LF settings such as North Darfur. Another limitation is that the survey did not include questions about residence and mobility. Because antibodies can reflect exposures years after they occur, seropositivity may not reflect local transmission status, particularly for mobile or displaced populations and adults. In the Darfur region, there are seasonally mobile ethnic groups and people who have been displaced owing to years of conflict. Without these additional data for OV, LF, and SCH, it was not possible to verify whether seropositivity by MBA indicated local exposure versus exposures experienced elsewhere, how many of the results were false-positives, or what programmatic actions to take based on the results. Further research is needed to guide OV, LF, and SCH program decisions in the interpretation of MBA data, especially in low- or nonendemic settings.

The results for Ov16 indicated little to no exposure to *O. volvulus*, with estimated seroprevalence significantly less than 1% overall and adult Ov16 seroprevalences significantly below the WHO start-MDA threshold of 2% in adults.^9^ Therefore, this study did not provide evidence to support initiation of ivermectin MDA for OV in these localities. This finding aligned with expectations from historical knowledge about OV distribution in Sudan and the absence of blackfly breeding sites in the region. By contrast, the relatively high seroprevalence estimates among children under 10 years of age in the study localities would not meet the WHO criteria to stop MDA in a formerly endemic area (significantly less than 0.1%).^10^^,^^44^ Other studies have assumed that Ov16 antibodies are long-lasting or that decreases in antibody density do not lead to complete seroreversion^45^^,^^46^; however, the older age groups in this study had very few seropositives and very low resulting estimates of seroprevalence. The lack of clear increase in Ov16 seropositivity with age in combination with the fact that MBA is thought to be less specific than ELISA owing to detecting all IgGs rather than IgG4 suggests that these could be false-positives, though we lack ELISA results for a true comparison.

Lymphatic filariasis results indicated a large range of seropositivity estimates depending on the antigen for Wb123 (3.6%), Bm14 (0.93%), and Bm33 (26.0%), in contradiction with the prior classification of these localities as nonendemic for LF. Lymphatic filariasis antibody responses develop earlier than antigenemia, and serosurveillance with sensitive tools may provide early warning of LF transmission.^29^ However, programmatic interpretation of the LF-associated antigen results from MBA is complex given the lack of international guidance for the use of LF serology, difficulty establishing thresholds to classify seropositivity and the resulting tradeoff between false-positives and false negatives, the known problem of cross-reactivity for Bm14 and Bm33, and the high level of discordance between the three antigens.^29^^,^^41^^,^^47^ This lack of concordance for seropositivity of the three antibodies is common in the literature,^29^^,^^39^^,^^48^^,^^49^ though studies comparing serology to antigenemia and microfilaremia have found that antigenemic and microfilaremic children tended to be positive for antibody responses to at least two and generally all three markers.^29^^,^^48^ Although composite variables requiring positive results to multiple LF antigens may improve specificity (Supplemental File 5), there is no standard algorithm, and we lack antigenemia and microfilaremia data for validation of the composite variable options in our study population. These data could be used as part of a larger meta-analysis to create guidance on the use of results from multi-antigen serological tests for LF.^50^ Ultimately, the discrepancies between antibody responses to the three antigens highlight the need for an algorithm to interpret LF serology results, more study of the antigen-specific antibody kinetics, and the identification of novel, more-specific markers to quantify *W. bancrofti* exposures, especially in low-endemicity settings where the positive predictive value will be low compared with higher endemicity areas.

The relative abundance of seropositivity to the three LF antigens is consistent with past MBA studies where Bm33 was more prevalent than Wb123 or Bm14.^39^^,^^48^ However, our results are unique in finding Wb123 more seroprevalent than Bm14. Previous studies have applied MBA technology for stop-MDA and post-treatment surveillance phases^48^^,^^51^ or general surveillance in LF-endemic countries.^38^^,^^39^ This appears to be the first study to apply MBA for LF serological assessment in historically nonendemic areas. All three localities included in this survey were classified as nonendemic for LF during the FMOH mapping surveys in 2016. Although Bm33 antibody responses are thought to suffer from cross-reactivity to other parasite exposures, Wb123 IgG4 responses are thought to be more specific.^23^^,^^29^ The overall estimated Wb123 seroprevalence of 3.6% in this study is disconcertingly high, though our seroprevalence estimates are likely limited by the lower specificity of using MBA to detect total IgG rather than IgG4. Similar findings have confounded interpretation in other presumed nonendemic areas where elevated levels of Wb123 were detected, though results from that study may be attributed in part to suboptimal performance of a commercial ELISA kit.^52^ The WHO guidelines for starting and stopping MDA for *W. bancrofti* are based on microfilaremia or CFA prevalence rather than serology, as antibody persistence may overestimate the number of active infections. Thus, no programmatic decisions can be taken on the results of this study alone. Instead, the program plans to conduct confirmatory “mini-transmission assessment surveys” remapping surveys to verify whether MDA is required in these three localities.^41^

Among SAC, the prevalence of antibody responses to SEA ranged from 3% to 6% and responses to Sm25 ranged from 11% to 16%, suggesting a history of exposure within the age group most often targeted by SCH control programs. Although programs typically monitor infection detected by microscopic detection of parasite eggs in stool or urine, recent analysis has demonstrated that seroprevalence is highly correlated with infection prevalence.^30^^,^^53^ It has also been shown that seroprevalence is more sensitive than parasite detection, particularly in low-prevalence settings.^53^ This may explain why the seroprevalence observed in this study was generally higher than the infection prevalence observed in these localities a few years earlier.^13^ Given the long-lived nature of IgG antibodies, it has also been suggested that PSAC may be a more appropriate age group in which to monitor current transmission and programmatic impact than SAC using serology.^30^^,^^53^ In this study, although the seroprevalence in PSAC was below 4% among all localities for either antigen, it did suggest that at least some transmission was occurring among young children in these settings. Given the observed heterogeneity of seroprevalence at the cluster level, further surveys that include traditional microscopy methodologies, focused on detecting hotspots of infection, may be needed to design efficient intervention strategies in this part of Sudan.^54^

Despite the advantages of serosurveys, key questions remain on the epidemiology of SCH in these localities. Data on location and characteristics of water bodies were not available, and thus it was not possible to determine the source of the infection in these localities. As traditional trachoma surveys do not map surface water, other tools would be needed to map snail habitats in suspected endemic settings for fine-scale intervention design.^55^ Collecting local knowledge about available water sources through focus group discussions or in-depth interviews with communities identified as having a higher seroprevalence may be one possible next step.

## CONCLUSION

The results of this survey supported the classification of El Seraif, Kotom, and Saraf Omrah localities, North Darfur State, as nonendemic for OV and prompted confirmatory remapping to determine LF transmission status in the study areas. This survey’s results, conducted just 2 years after a nationwide survey, also confirmed transmission of the *Schistosoma* spp. in these localities. This suggests that more targeted SCH assessments are likely needed to confirm the need for MDA. Neglected tropical disease elimination programs rely on epidemiological surveillance data such as those provided in this study, but surveys are resource intensive and therefore difficult to undertake regularly. Cross-program integration of data collection and analysis is a key solution to these limitations and is recommended in the second pillar of the WHO’s NTD road map: “Intensify cross-cutting approaches.”^7^ Although interpretations of the results for OV, LF, and SCH antigens are limited by the lack of WHO guidelines on MBA serology data in the absence of validation against accepted programmatic tests, the results from this integrated multiplex trachoma survey provided epidemiological data to other NTD programs in Sudan at minimal additional cost. Further research on new antigens and guidelines on the interpretation of results from MBA for OV, LF, and SCH, particularly in low-endemicity areas, are required to maximize the potential of this integrated testing.

## Supplemental Materials

10.4269/ajtmh.23-0760 Supplemental Materials

10.4269/ajtmh.23-0760 Supplemental Materials

10.4269/ajtmh.23-0760 Supplemental Materials

10.4269/ajtmh.23-0760 Supplemental Materials

10.4269/ajtmh.23-0760 Supplemental Materials

10.4269/ajtmh.23-0760 Supplemental Materials
