# Supplementary figures and images for: Integrated Serosurveillance for Onchocerciasis, Lymphatic Filariasis, and Schistosomiasis in North Darfur, Sudan
Source: Am J Trop Med Hyg. 2024 Jun 25;111(3 Suppl):58–68. doi: 10.4269/ajtmh.23-0760 (PMC11376112; doi:10.4269/ajtmh.23-0760)

A

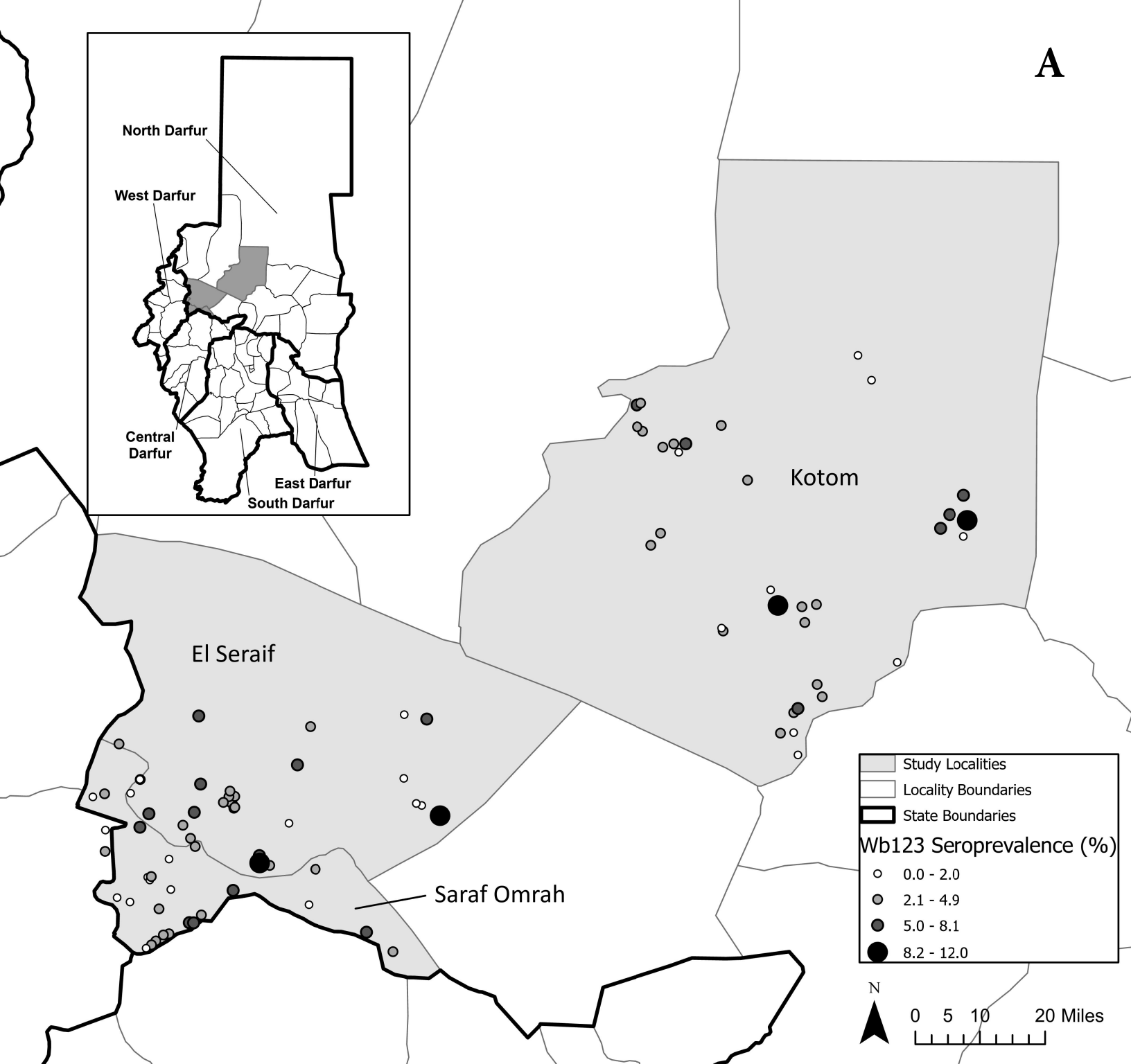

**B**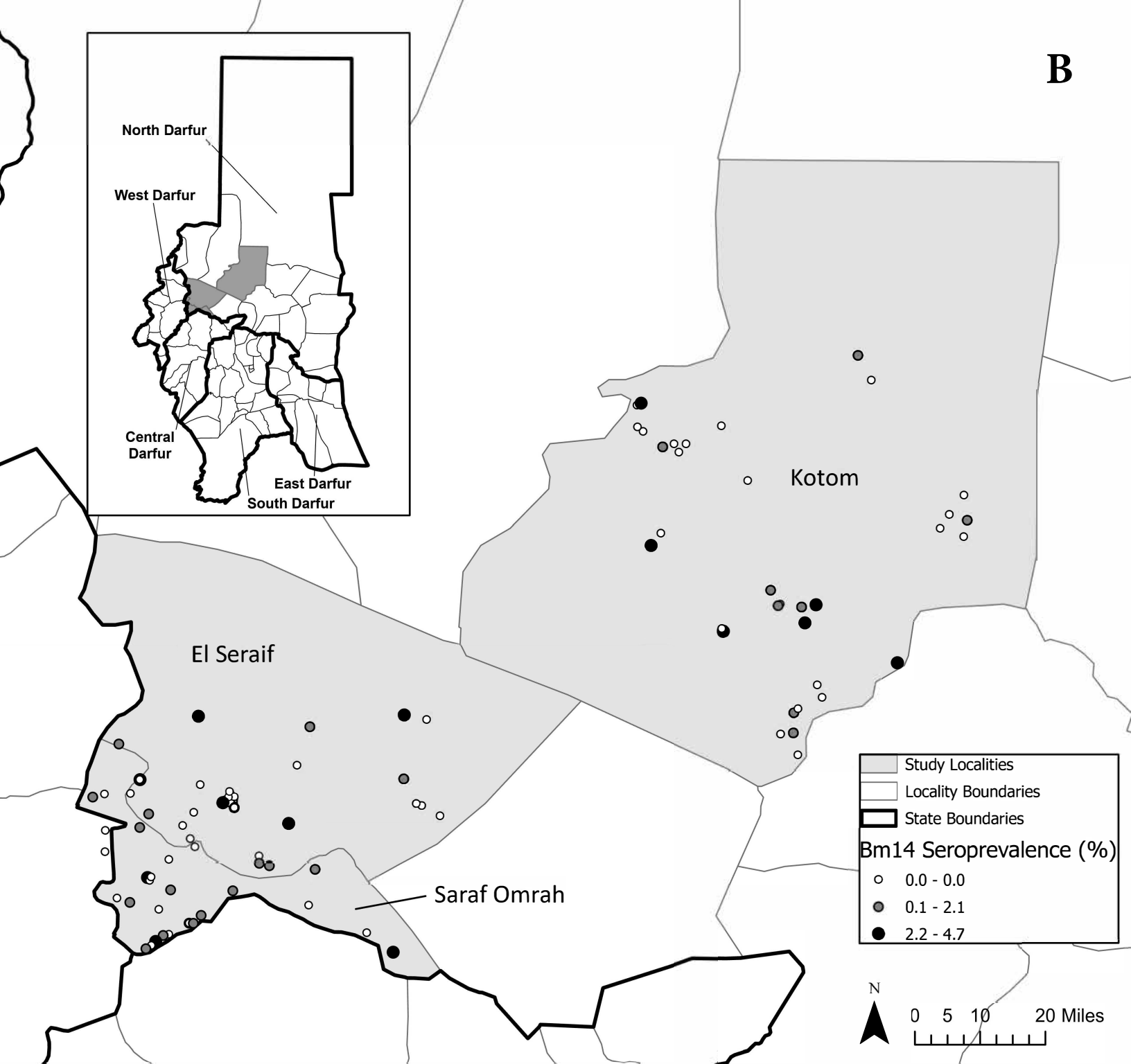

C

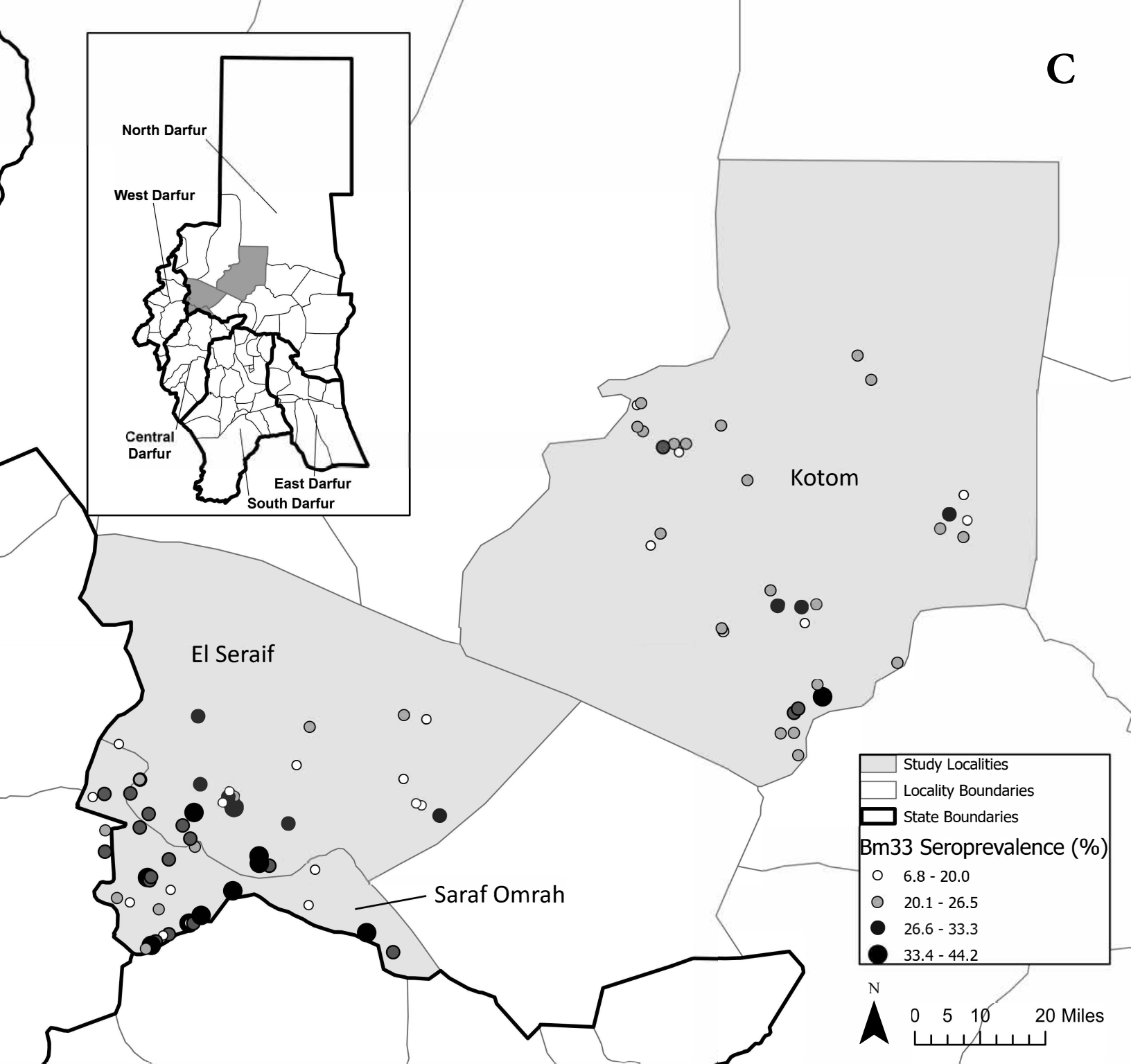

Supplement: Supplemental Materials [file tpmd230760.SD1.pdf]

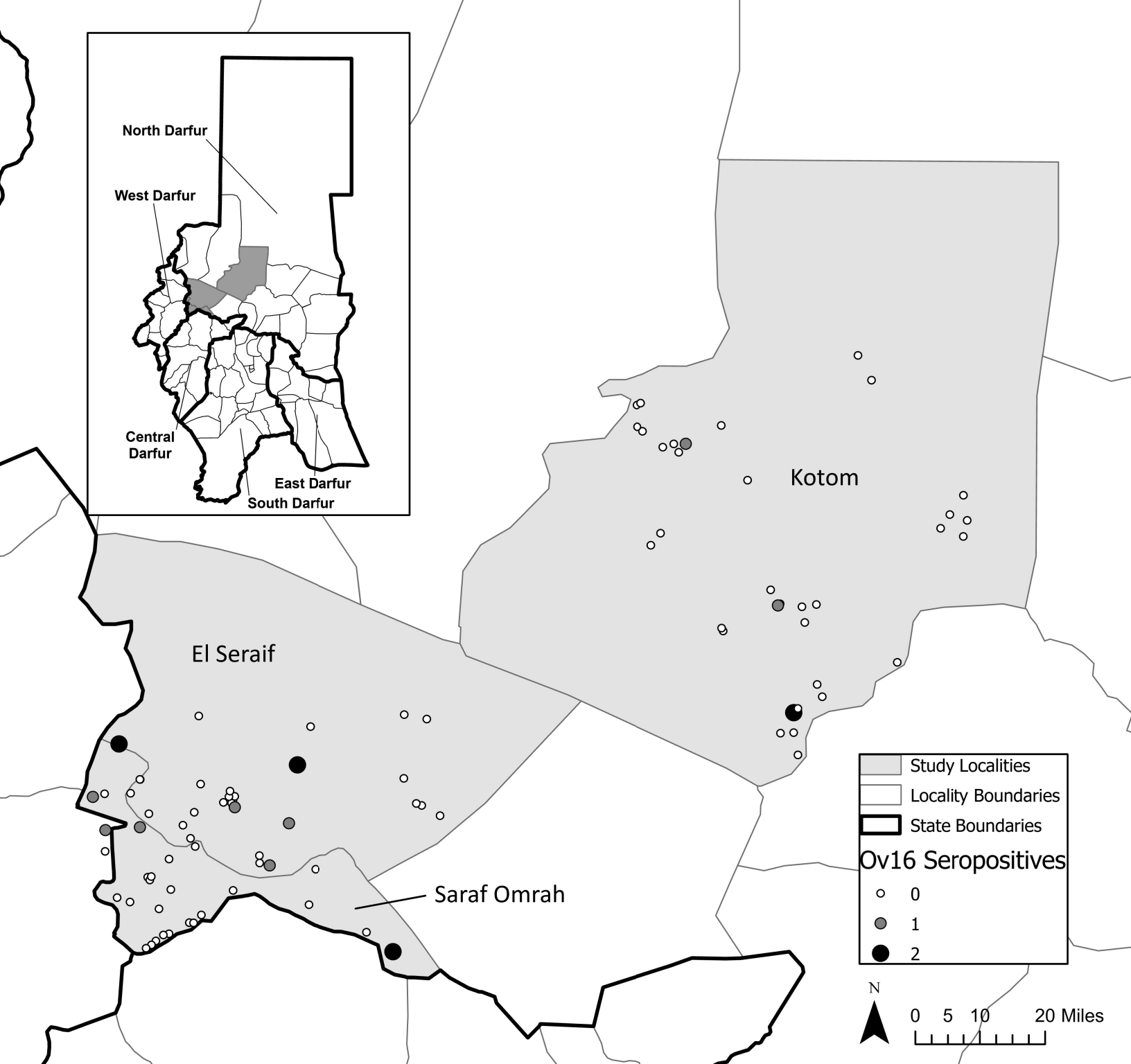

Supplement: Supplemental Materials [file tpmd230760.SD2.pdf]

A

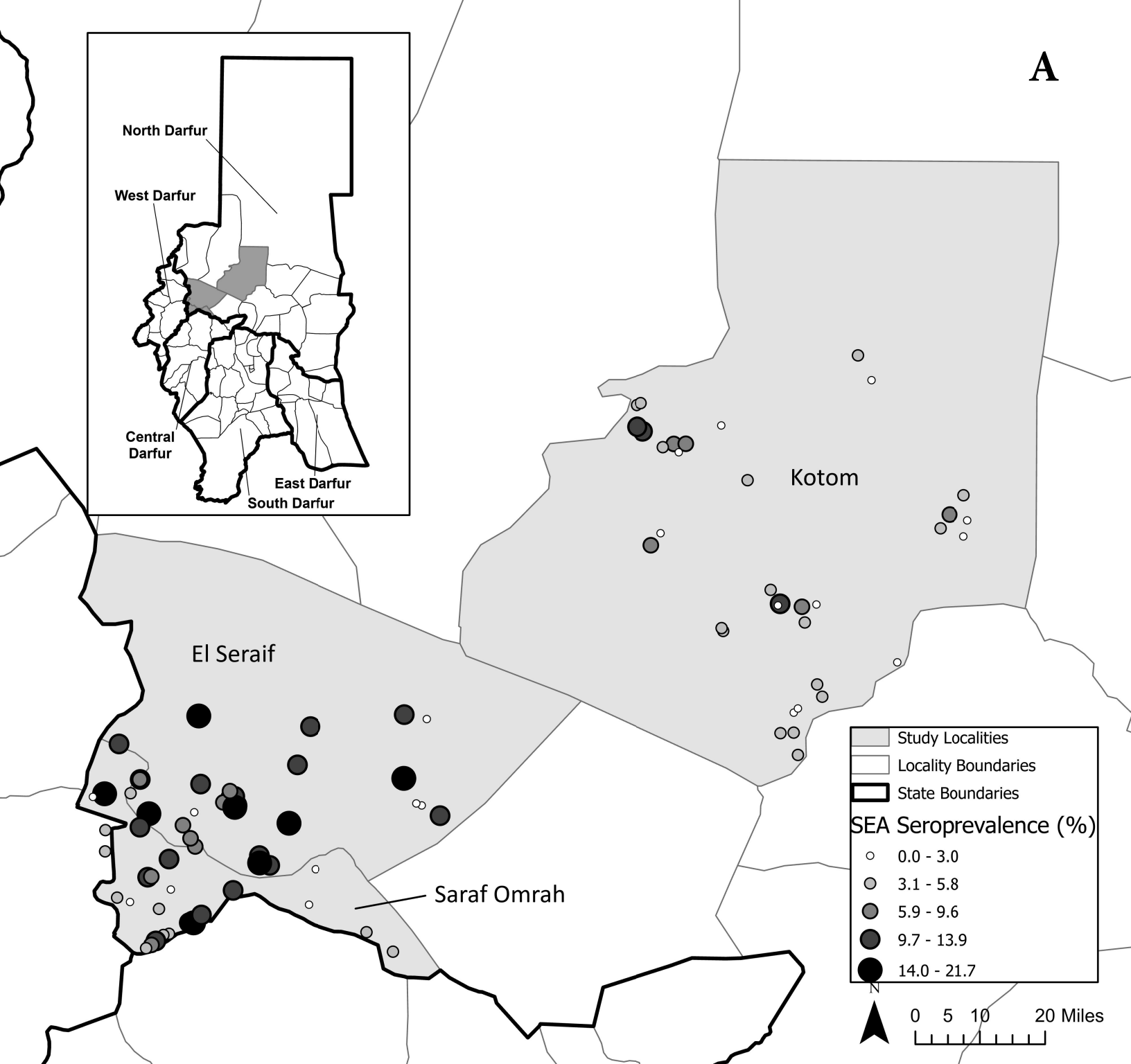

**B**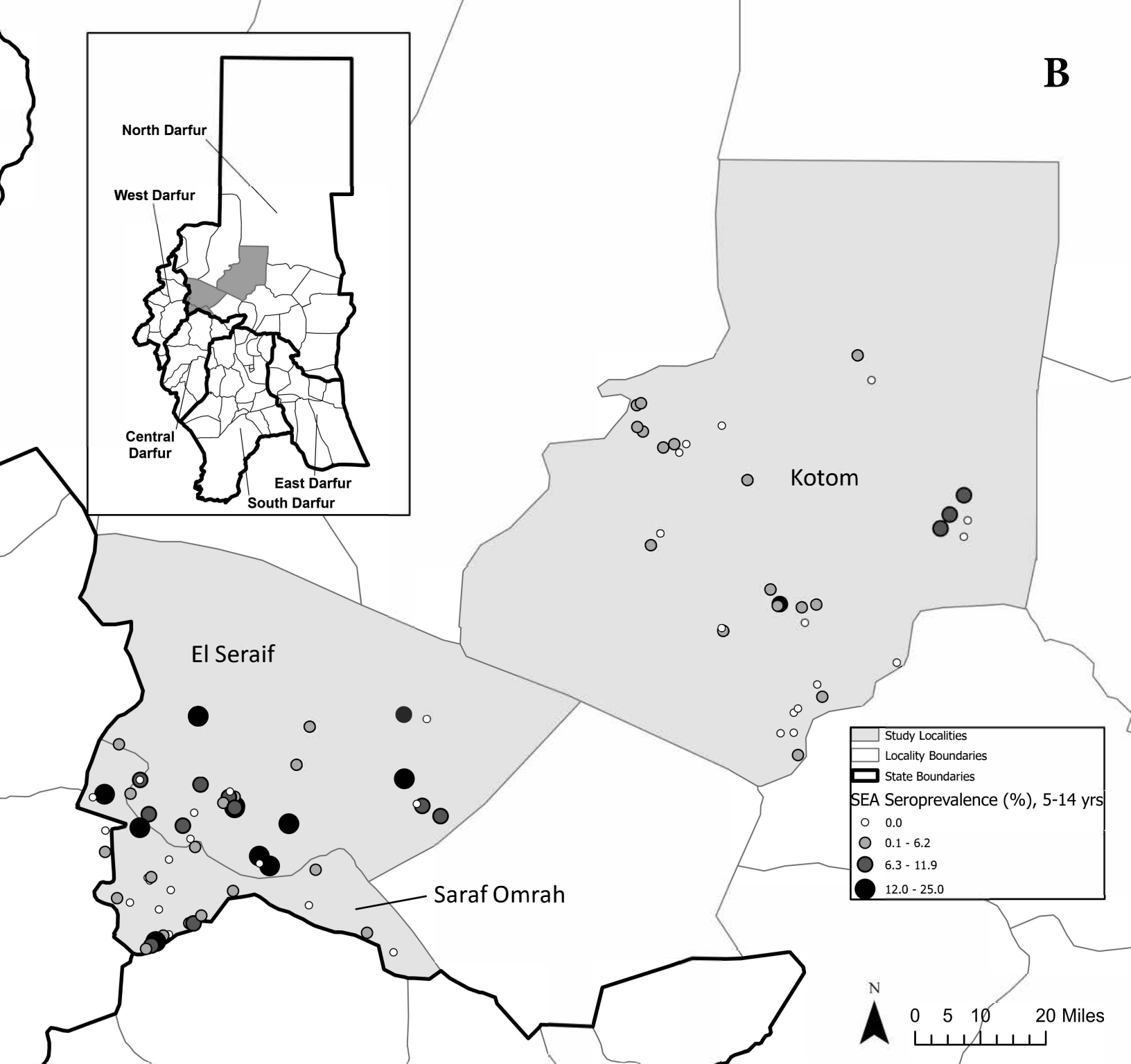

C

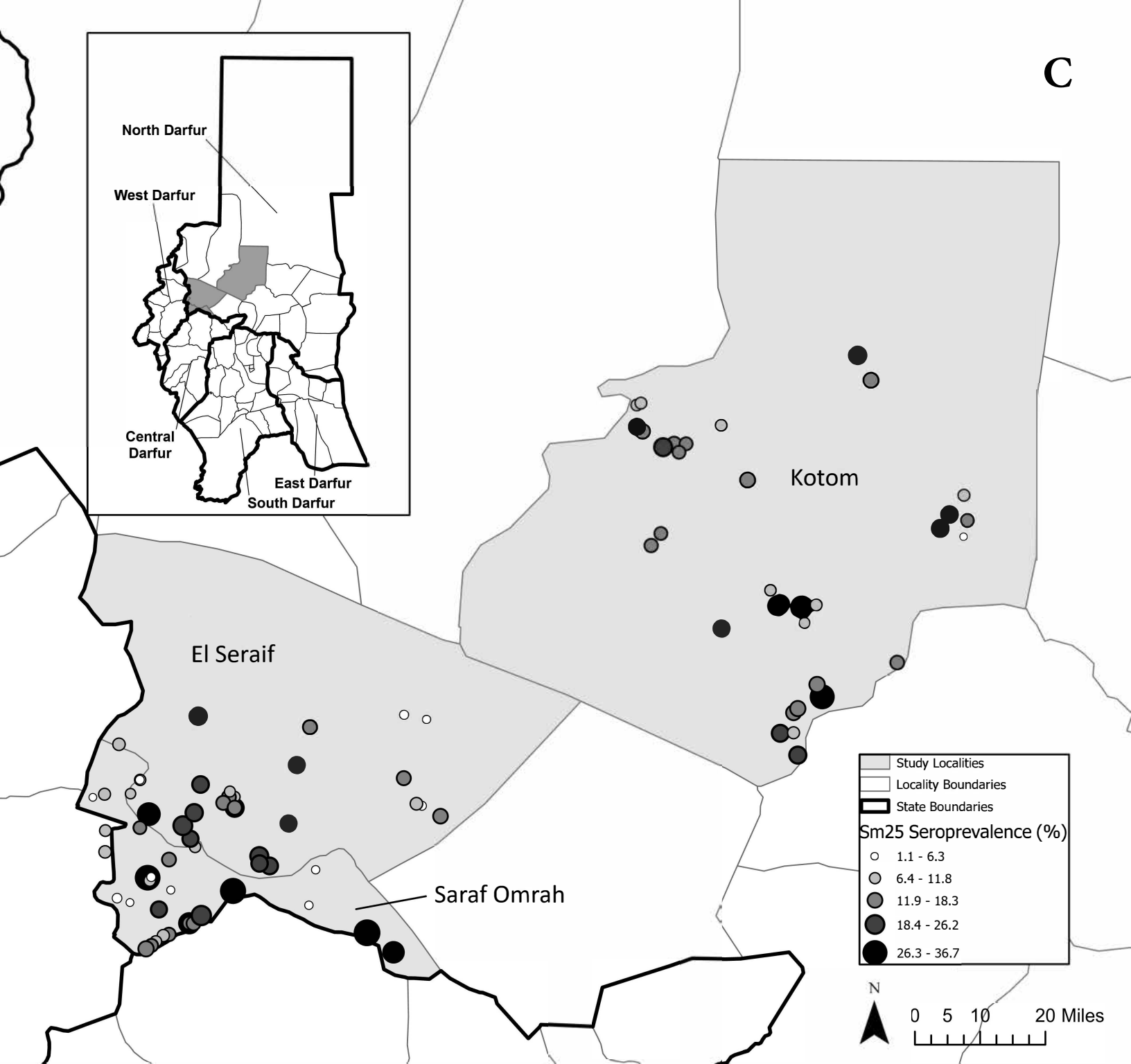

D

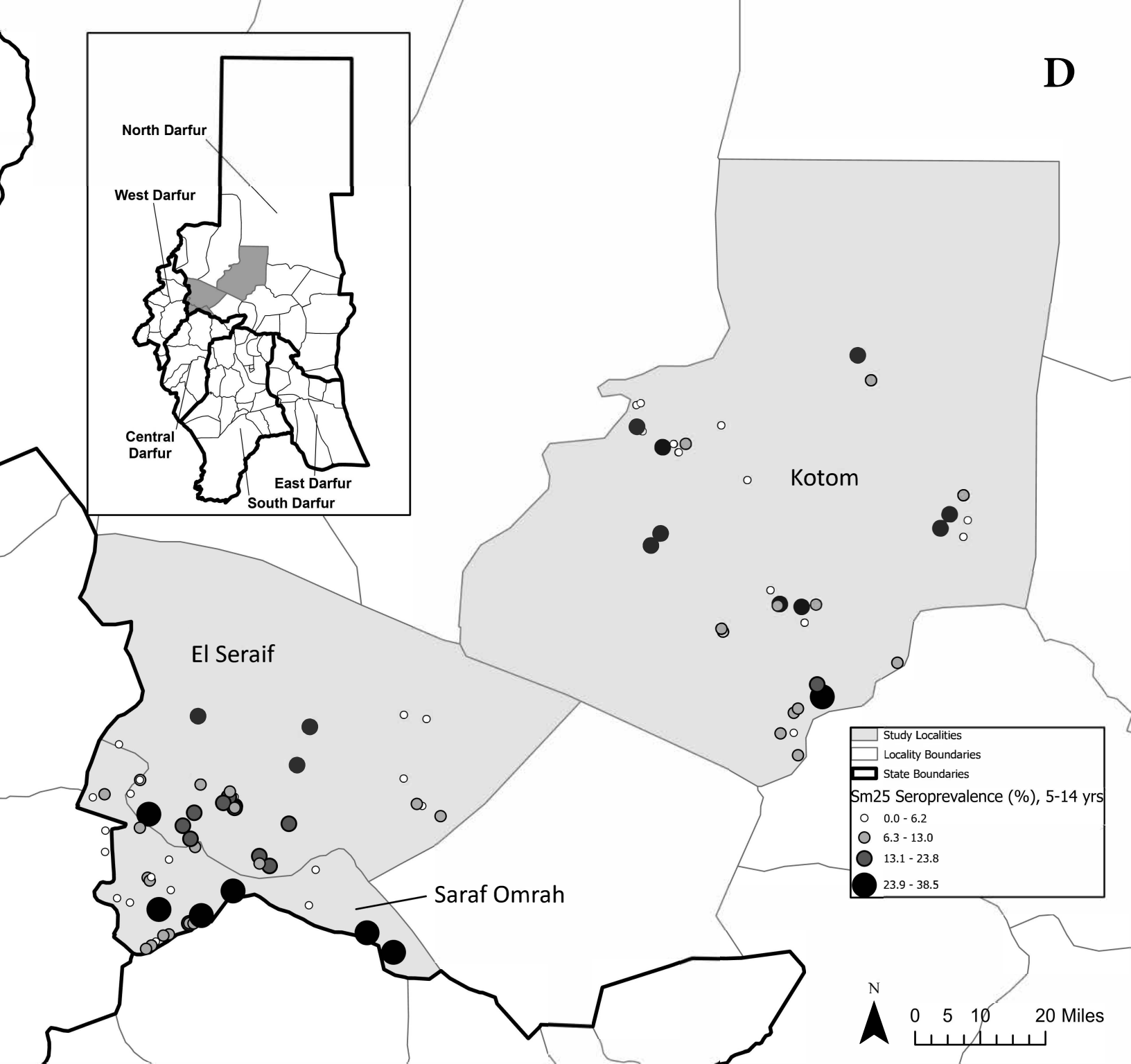

Supplement: Supplemental Materials [file tpmd230760.SD6.pdf]
