# Supplementary material for: Integrated Serosurveillance for Onchocerciasis, Lymphatic Filariasis, and Schistosomiasis in North Darfur, Sudan
Source: Am J Trop Med Hyg. 2024 Jun 25;111(3 Suppl):58–68. doi: 10.4269/ajtmh.23-0760 (PMC11376112; doi:10.4269/ajtmh.23-0760)
Supplement: Supplemental Materials [file tpmd230760.SD3.pdf]

Supplemental File S1. Seroconversion and seroreversion[a] rates per year by locality estimated from a simple reversible catalytic model for filiarial disease and schistosomiasis antibodies by multiplex bead array (MBA) assay, North Darfur, Sudan, 2019-2020

	El Seraif		Kotom		Saraf Omrah	
**Antigen**	**SCR (95% CI)**	**SRR (95% CI)**	**SCR (95% CI)**	**SRR (95% CI)**	**SCR (95% CI)**	**SRR (95% CI)**
Ov16	0.0043 (1.7e^{-6} – 11.2)	2.46 (9.5e^{-4} – 6414.6)	0.0040 (3.1e^{-7} – 52.3)	2.80 (2.2e^{-4} – 36,176.5)	5.40e^{-4} (4.6e^{-5} – 0.0065)	0.15 (0.0052 – 4.47)
Wb123	0.0070 (0.0039 – 0.013)	0.12 (0.050 – 0.27)	0.0095 (0.0049 – 0.018)	0.20 (0.089 – 0.44)	0.0063 (0.0031 – 0.013)	0.14 (0.050 – 0.37)
Bm14	0.011 (1.1e^{-4} – 1.22)	1.23 (0.010 – 148.4)	0.0019 (6.3e^{-4} – 0.0058)	0.18 (0.049 – 0.69)	0.0015 (5.9e^{-4} – 0.0039)	0.13 (0.036 – 0.45)
Bm33	0.075 (0.060 – 0.095)	0.16 (0.12 – 0.22)	0.071 (0.054 – 0.094)	0.18 (0.13 – 0.26)	0.095 (0.074 – 0.12)	0.21 (0.16 – 0.29)
LF composite[b]	0.003 (0.001 – 0.004)	0.066 (0.026 – 0.168)	0.002 (0.001 – 0.004)	0.078 (0.030 – 0.202)	0.004 (0.002 – 0.009)	0.198 (0.064 – 0.609)
SEA	0.012 (0.010 – 0.015)	0.042 (0.027 – 0.064)	0.0039 (0.0024 – 0.0062)	0.043 (0.019 – 0.100)	0.0050 (0.0039 – 0.0065)	0.0082 (0.0017 – 0.0393)
Sm25	0.018 (0.015 – 0.021)	0.040 (0.027 – 0.058)	0.014 (0.012 – 0.017)	0.027 (0.018 – 0.042)	0.014 (0.011 – 0.017)	0.030 (0.018 – 0.049)

CI = Confidence interval; SCR = Seroconversion rate; SRR = Seroreversion rate

[a] Estimation of seroreversion rates is limited by the cross-sectional nature of the data. All models assumed a constant force of infection over time.

[b] The composite LF variable included subjects as seropositive only if they were seropositive to at least 2 of the LF antigens Wb123, Bm14, and Bm33
