# Supplementary material for: Integrated Serosurveillance for Onchocerciasis, Lymphatic Filariasis, and Schistosomiasis in North Darfur, Sudan
Source: Am J Trop Med Hyg. 2024 Jun 25;111(3 Suppl):58–68. doi: 10.4269/ajtmh.23-0760 (PMC11376112; doi:10.4269/ajtmh.23-0760)
Supplement: Supplemental Materials [file tpmd230760.SD4.pdf]

Supplemental File S5. Seropositivity by various composites of the 3 lymphatic filariasis antigens Wb123, Bm14, and Bm33 by locality per multiplex bead array assay, North Darfur Cross-sectional Survey 2019-2020

	El Seraif			Kotom			Saraf Omrah		
	n + / N tested	Seroprevalence (95% CI)	p-value[1]	n + / N tested	Seroprevalence (95% CI)	p-value[1]	n + / N tested	Seroprevalence (95% CI)	p-value[1]
LF seropositivity to all 3 antigens									
Total	2/2848	0.04% (0.01 – 0.16%)		0/2781	0% (n/a)		1/2693	0.04% (0.01 – 0.27%)	
Sex									
Women	1/1680	0.02% (0.003 – 0.13%)		0/1699	0% (n/a)		1/1643	0.06% (0.01 – 0.44%)	
Men	1/1168	0.06% (0.01 – 0.44%)	0.37	0/1082	0% (n/a)	n/a	0/1050	0% (n/a)	0.42
Age									
1-9 y	0/1275	0% (n/a)		0/1127	0% (n/a)		0/1272	0% (n/a)	
10-19 y	1/627	0.12% (0.02 – 0.83%)		0/483	0% (n/a)		0/554	0% (n/a)	
Adults ≥20 y	1/946	0.04% (0.01 – 0.24%)	0.47	0/1171	0% (n/a)	n/a	1/867	0.12% (0.02 – 0.86%)	**<0.001**
LF seropositivity to any 2 or all 3 antigens									
Total	58/2848	2.0% (1.3 – 3.1%)		42/2781	1.6% (1.1 – 2.3%)		41/2693	1.6% (1.1 – 2.2%)	
Sex									
Women	33/1680	2.0% (1.2 – 3.3%)		32/1699	2.0% (1.3 – 3.0%)		29/1643	1.9% (1.2 – 2.9%)	
Men	25/1168	1.9% (1.2 – 3.2%)	0.83	10/1082	1.1% (0.4 – 2.5%)	0.22	12/1050	1.1% (0.6 – 2.0%)	0.16
Age									
1-9 y	13/1275	0.6% (0.3 – 1.4%)		7/1127	0.5% (0.2 – 1.3%)		14/1272	1.1% (0.6 – 2.1%)	
10-19 y	16/627	2.6% (1.0 – 6.5%)		8/483	1.7% (0.8 – 3.8%)		12/554	2.0% (1.1 – 3.8%)	
Adults ≥20 y	29/946	3.4% (2.2 – 5.3%)	**<0.001**	27/1171	2.7% (1.7 – 4.2%)	**0.03**	15/867	1.9% (1.1 – 3.3%)	0.19
Bm33 and Wb123 seropositive									
Total	43/2848	1.5% (0.91 – 2.4%)		38/2781	1.4% (0.97 – 2.1%)		34/2693	1.3% (0.90 – 2.0%)	
Sex									
Women	23/1680	1.6% (0.86 – 3.0%)		29/1699	1.8% (1.1 – 2.9%)		23/1643	1.5% (0.92 – 2.5%)	
Men	20/1168	1.3% (0.80 – 2.1%)	0.53	9/1082	0.85% (0.40 – 1.8%)	0.14	11/1050	1.0% (0.56 – 2.0%)	0.35
Age									
1-9 y	10/1275	0.43% (0.19 – 0.98%)		7/1127	0.51% (0.20 – 1.3%)		12/1272	1.1% (0.55 – 2.1%)	
10-19 y	10/627	2.0% (0.64 – 6.3%)		5/483	0.81% (0.32 – 2.0%)		10/554	1.6% (0.72 – 3.4%)	
Adults ≥20 y	23/946	2.6% (1.6 – 4.2%)	**0.003**	26/1171	2.6% (1.6 – 4.1%)	**0.005**	12/867	1.6% (0.86 – 2.9%)	0.23
Bm33 and Bm14 seropositive									
Total	16/2848	0.47% (0.24 – 0.92%)		4/2781	0.20% (0.07 – 0.53%)		8/2693	0.25% (0.12 – 0.55%)	
Sex									
Women	11/1680	0.41% (0.18 – 0.94%)		3/1699	0.20% (0.06 – 0.60%)		7/1643	0.39% (0.17 – 0.88%)	
Men	5/1168	0.56% (0.23 – 1.3%)	0.57	1/1082	0.20% (0.03 – 1.4%)	0.97	1/1050	0.04% (0.01 – 0.26%)	0.01
Age									
1-9 y	3/1275	0.21% (0.04 – 1.1%)		0/1127	0% (n/a)		2/1272	0.05% (0.01 – 0.20%)	
10-19 y	7/627	0.71% (0.28 – 1.8%)		3/483	0.91% (0.29 – 2.8%)		2/554	0.44% (0.10 – 1.8%)	
Adults ≥20 y	6/946	0.68% (0.28 – 1.6%)	0.29	1/1171	0.12% (0.02 – 0.86%)	**<0.001**	4/867	0.43% (0.14 – 1.3%)	0.19
Wb123 and Bm14 seropositive									

Total	3/2848	0.08% (0.02 – 0.28%)		0/2781	0% (n/a)		1/2693	0.04% (0.01 – 0.27%)	
Sex									
Women	1/1680	0.02% (0.003 – 0.13%)		0/1699	0% (n/a)		1/1643	0.06% (0.01 – 0.44%)	
Men	2/1168	0.17% (0.04 – 0.71%)	**0.04**	0/1082	0% (n/a)	n/a	0/1050	0% (n/a)	0.42
Age									
1-9 y	0/1275	0% (n/a)		0/1127	0% (n/a)		0/1272	0% (n/a)	
10-19 y	1/627	0.12% (0.02 – 0.83%)		0/483	0% (n/a)		0/554	0% (n/a)	
Adults ≥20 y	2/946	0.17% (0.04 – 0.82%)	0.06	0/1171	0% (n/a)	n/a	1/867	0.12% (0.02 – 0.86%)	**<0.001**

Bolded p-values indicate statistical significance at <0.05.

CI = Confidence interval; LF = Lymphatic filariasis; PSAC = Pre-school aged children; SAC = School-aged children

[1] p-values were calculated from Chi-squared tests for sex and t-tests for age as a continuous variable, accounting for the survey weights using svy commands in Stata.
