# Supplementary material for: Integrated Serosurveillance for Onchocerciasis, Lymphatic Filariasis, and Schistosomiasis in North Darfur, Sudan
Source: Am J Trop Med Hyg. 2024 Jun 25;111(3 Suppl):58–68. doi: 10.4269/ajtmh.23-0760 (PMC11376112; doi:10.4269/ajtmh.23-0760)
Supplement: Supplemental Materials [file tpmd230760.SD5.pdf]

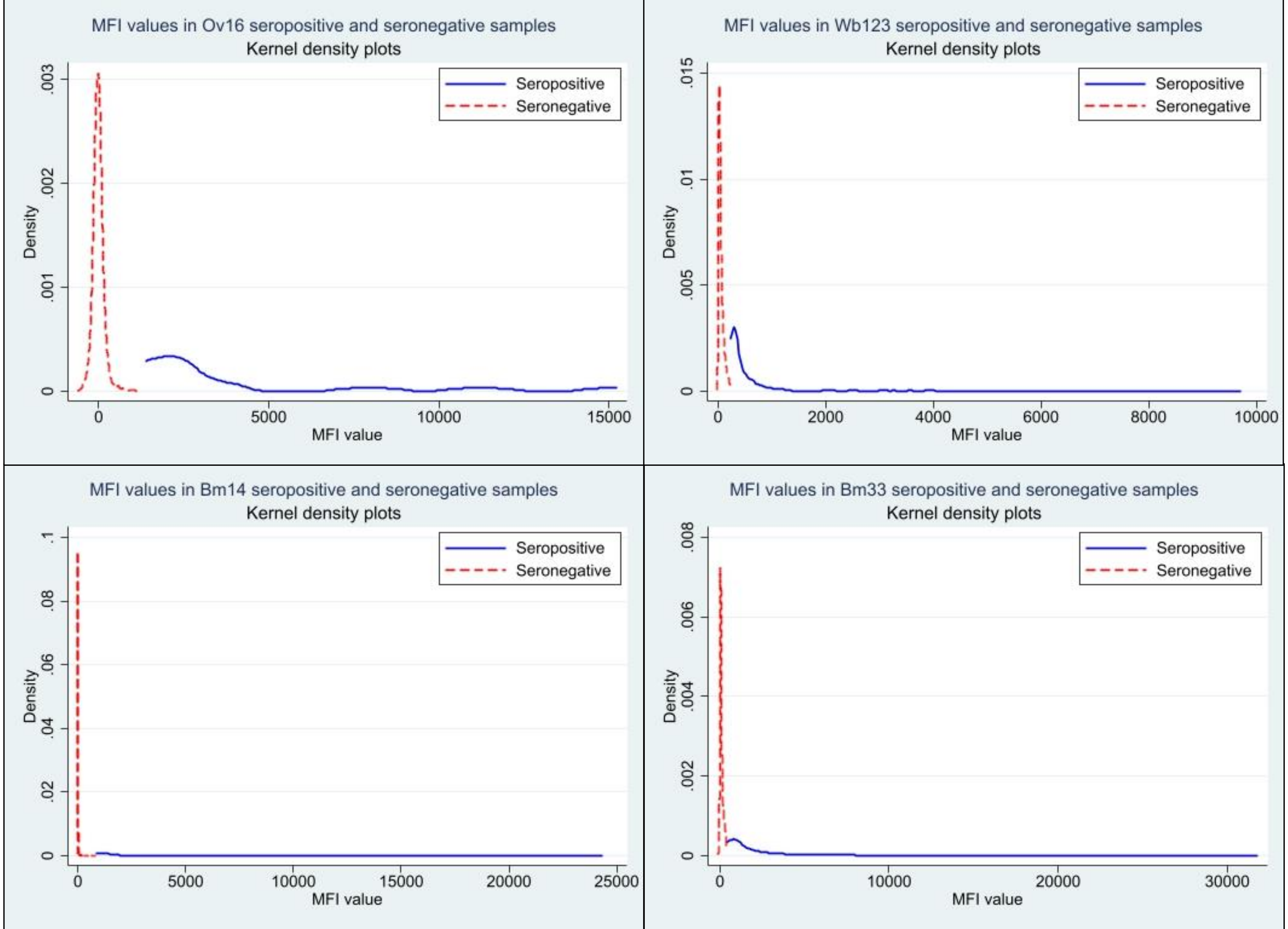

Supplemental File 2. Kernel density plots of median fluorescence intensity (MFI) values for individuals classified as seropositive vs. seronegative for (A) Ov16, (B) Wb123, (C) Bm14, and (D) Bm33
